# Vitamin D Actions on CD4^+^ T Cells in Autoimmune Disease

**DOI:** 10.3389/fimmu.2015.00100

**Published:** 2015-03-18

**Authors:** Colleen Elizabeth Hayes, Shane L. Hubler, Jerott R. Moore, Lauren E. Barta, Corinne E. Praska, Faye E. Nashold

**Affiliations:** ^1^Department of Biochemistry, College of Agricultural and Life Sciences, University of Wisconsin-Madison, Madison, WI, USA; ^2^Department of Statistics, College of Letters and Sciences, University of Wisconsin-Madison, Madison, WI, USA

**Keywords:** vitamin D, CD4-positive T lymphocytes, autoimmune diseases, multiple sclerosis, type 1 diabetes

## Abstract

This review summarizes and integrates research on vitamin D and CD4^+^ T-lymphocyte biology to develop new mechanistic insights into the molecular etiology of autoimmune disease. A deep understanding of molecular mechanisms relevant to gene–environment interactions is needed to deliver etiology-based autoimmune disease prevention and treatment strategies. Evidence linking sunlight, vitamin D, and the risk of multiple sclerosis and type 1 diabetes is summarized to develop the thesis that vitamin D is the environmental factor that most strongly influences autoimmune disease development. Evidence for CD4^+^ T-cell involvement in autoimmune disease pathogenesis and for paracrine calcitriol signaling to CD4^+^ T lymphocytes is summarized to support the thesis that calcitriol is sunlight’s main protective signal transducer in autoimmune disease risk. Animal modeling and human mechanistic data are summarized to support the view that vitamin D probably influences thymic negative selection, effector Th1 and Th17 pathogenesis and responsiveness to extrinsic cell death signals, FoxP3^+^CD4^+^ T-regulatory cell and CD4^+^ T-regulatory cell type 1 (Tr1) cell functions, and a Th1–Tr1 switch. The proposed Th1–Tr1 switch appears to bridge two stable, self-reinforcing immune states, pro- and anti-inflammatory, each with a characteristic gene regulatory network. The bi-stable switch would enable T cells to integrate signals from pathogens, hormones, cell–cell interactions, and soluble mediators and respond in a biologically appropriate manner. Finally, unanswered questions and potentially informative future research directions are highlighted to speed delivery of etiology-based strategies to reduce autoimmune disease.

## Introduction

Autoimmune diseases afflict ~50 million Americans and contribute >$100 billion to US health care costs (1). The global burden has risen with the near tripling in the last half-century of multiple sclerosis (MS) (2, 3), type 1 diabetes (T1D) (4), and other autoimmune diseases. A deep understanding of disease mechanisms will be needed to deliver etiology-based strategies to reverse this vexing trend. Indeed, “functional and mechanistic work on the molecular etiology of disease remains one of the major challenges in modern biology” (5).

This review highlights recent advances in vitamin D and T-lymphocyte biology in an effort to harness vitamin D’s potential to reduce the impact of autoimmune diseases. Gene–environment interactions, sunlight and vitamin D, and T lymphocytes as autoimmune disease initiators and vitamin D targets are discussed to explain the rationale for reviewing vitamin D mechanisms in T cells. Research on vitamin D regulation of thymocyte selection, Th1 and Th17 cells, T-cell programed cell death, and T-regulatory (Treg) cells is summarized and integrated into model mechanisms. Finally, unanswered questions relating to vitamin D mechanisms in CD4^+^ T cells are highlighted to promote further research that may lead to a deeper understanding of autoimmune disease molecular etiology.

## Genes, Sunlight, Vitamin D, and T Lymphocytes

### Autoimmune diseases

Autoimmune diseases represent a failure of self-identification leading to an immune-mediated assault on host tissues. More than 100 autoimmune syndromes exist (1). We drew mainly on MS and T1D research because intensive investigation has generated detailed insights into vitamin D mechanisms in these diseases and provided valuable guidance for research on other autoimmune diseases. Other autoimmune diseases are included where robust mechanistic data exist. A recent chapter (6) and a review (7) have summarized vitamin D mechanisms in autoimmune diseases more generally.

Multiple sclerosis and T1D have distinct target organs, genetic risk factors, onset ages, and female to male ratios, but target organ-specific T cells as initiators unite these diseases. MS is the leading cause of non-traumatic neurological disability in young adults. It results from an autoimmune attack on the axon–myelin unit (8). Neurological dysfunction in MS is attributed to focal demyelinated lesions in the central nervous system (CNS). The neurodegenerative process is believed to occur sub-clinically before the disease is typically diagnosed in the third decade of life. Most MS patients have a relapsing-remitting form of the disease, and among them, women outnumber men 3:1.

Type 1 diabetes is a common chronic disease of childhood with an onset typically between ages 6 and 15. Boys with T1D slightly outnumber girls. T1D results from an autoimmune attack on insulin-producing pancreatic β-cells (9). Metabolic dysfunction in T1D is attributed to β-cell destruction and loss of insulin production and blood glucose control. The β-cell degenerative process is also believed to occur sub-clinically before a T1D diagnosis, when as much as 70% of β-cell mass has been destroyed and insulin supply no longer meets demand (10). We searched for analogies between MS and T1D that might reveal over-arching environmental influences on T lymphocytes in autoimmunity.

### Gene–environment interactions in autoimmune disease

Interacting genetic, environmental, and hormonal influences drive autoimmune disease. There is a weak genetic component in autoimmune disease susceptibility. MS risk increases a modest 2–6% among first-degree biological relatives of an MS index case (11), but candidate risk loci are of such modest aggregate influence that genetic burden scores cannot accurately predict disease (12, 13). Autoimmune disease discordance between monozygotic twins is generally between 60 and 75% and cannot be explained by somatic mosaicism in MS (14), T1D (15), Crohn’s disease (CD) (16, 17), or systemic lupus erythematosus (SLE) (18). The low penetrance of candidate risk loci, high monozygotic twin discordance rates, and rapidly rising disease incidence rates underscore the hypothesis that *an autoimmune disease phenotype emerges when modifiable environmental stressors act on a disease-susceptible genotype, and exposure to at least one environmental stressor is increasing*. The key questions are (i) what are the dominant, modifiable environmental stressors, (ii) by what mechanisms do they interact with susceptibility genes to propel the disease process, and (iii) can mechanistic knowledge of gene–environment interactions be exploited to craft effective, etiology-based disease prevention and treatment strategies.

### Sunlight exposure and risk of MS and T1D disease

There is a large, latitude-linked, non-transmissible environmental component acting in a female-biased manner at the population level that determines whether the MS phenotype emerges from an MS-susceptible genotype (19, 20). Supporting this interpretation are the gradient in MS prevalence as a function of latitude (21, 22), alteration of MS risk by migration (23), and the equivalent MS risk between non-biological relatives of an MS case and the general population (24). The latitudinal gradient has dissipated as incidence has risen in low latitude regions. Peak MS prevalence was ~130/10^5^ population in 1960 (21) and is now ~400/10^5^ (22). Young women have borne the brunt of rising MS incidence (25, 26). The rise in female MS incidence implies a significant proportion of new female MS cases may be preventable (27).

Low sunlight exposure is postulated to be the major latitude-linked component in MS risk (21). Ultraviolet (UV) irradiance varies 400-fold with latitude (28), correlating inversely with the ~400-fold variation in MS prevalence (22). UV irradiance also varies seasonally at high latitudes, where increases in MS disease activity have been noted lagging seasonal declines in ambient UV light (29–31). Furthermore, childhood and occupational sunlight exposure correlated inversely with MS disease risk (32–35) and mortality (36).

Low sunlight exposure is also postulated to be a major component in T1D risk. Globally, T1D incidence varied ~350-fold (37) correlating inversely with the 400-fold latitudinal variation in UV irradiance (28). Global T1D incidence is increasing, while the proportion of T1D patients with the major *HLA* risk genotype is decreasing (13), implicating a modifiable environmental factor. T1D onset peaked between October and January and reached a nadir between June and August in the northern hemisphere, with a reverse pattern in the southern hemisphere (38). This correlation disappeared after adjustment for latitude. The inverse correlation between ambient winter UV radiation and T1D (*r* = −0.80) (39) was nearly as robust as that for MS (*r* = −0.9) (21). Thus, ambient UV irradiance is the leading candidate for the large, latitude-linked environmental risk factor in both MS and T1D (40, 41).

The correlative data are consistent with but do not prove that low sunlight exposure is a major component in autoimmune disease risk. Other environmental risk factors have been proposed (42), but they have markedly smaller effect sizes. For example, symptomatic Epstein-Barr virus (EBV) infection in adolescence (mononucleosis), the strongest of the environmental risk factors that appear to act independently of latitude, season, and UV light (43), correlates with a two to threefold increase in MS risk (44). In sharp contrast, residence in a low ambient UV light region correlates with a >100-fold increase in MS risk (22). Whether EBV infection and UV light are completely independent risk factors is currently debated (45). As discussed below, genetic data suggest the *VDR* gene influences HLA-DRB1 presentation of peptides to CD4^+^ T lymphocytes, and structural data show pathogenic T cells did not distinguish a DRB1*1501-restricted myelin peptide from a DRB5*0101-restricted EBV peptide suggesting a molecular mimicry mechanism underlying pathogenesis. In summary, the UV-linked component exerts its influence on multiple autoimmune diseases and on populations with disparate ancestries, distinct dietary and smoking habits, and dissimilar exposures to infectious and commensal organisms. These qualities of strength and universality provide the rationale for close investigation of this component’s identity and mechanisms.

### Vitamin D and transduction of sunlight’s protective signals in MS and T1D

Vitamin D_3_ and its hormonal form, 1α,25-dihydroxyvitamin D_3_ (calcitriol) have been proposed as the major biological transducers of sunlight’s protective signals in autoimmune disease due to calcitriol’s ability to selectively regulate T-cell-mediated autoimmune responses (46). Evidence supporting this hypothesis has been reviewed (6, 7, 42, 47, 48). Persuasive evidence for vitamin D as sunlight’s signal transducer derives from genetic linkage studies. Rare loss-of-function mutations in the *CYP27B1* gene correlated with a significantly increased autoimmune disease risk. This association was first reported for T1D (49–54), Addison’s disease (55), Hashimoto’s thyroiditis, and Graves’ disease (56). It was subsequently reported for MS (57–60). In rare multi-incident MS families, 35 of 35 cases inherited one defective *CYP27B1* allele, an inheritance pattern with small odds (one in a billion) of occurring by chance (58). Because *CYP27B1* mutations are highly penetrant but exceedingly rare, they do not contribute genetic risk in the vast majority of disease cases. In fact, genome-wide association studies (GWAS) and some case–control studies did not detect an association between *CYP27B1* variants and MS or T1D (61–65). However, the replicated positive genetic findings indelibly mark calcitriol synthesis as a key determinant of MS and T1D risk.

Correlations between *VDR* alleles and MS susceptibility have also been reported (66–68). An early study found a *VDR* and MS association in patients who carried the high-risk HLA-DRB1*1501 allele (69). Later research identified a putative vitamin D-responsive element (VDRE; see below) in the HLA-DRB1*1501 promoter (70). The *VDR* association data have been inconsistent between populations, and some *VDR* polymorphisms studied do not have known functional impacts. The *VDR Fok*I polymorphism is an exception; *VDR*^F^ (without the *Fok*I site) encodes a 424 amino acid protein with higher transcriptional activity than the 427 amino acid protein encoded by *VDR*^f^ (a *Fok*I site in the first ATG codon) (71, 72). The less active VDR^f^ protein was associated with higher serum 25-hydroxyvitamin D (25-OHD) levels, lower MS risk, and lower MS disability (73–76). Another exception is the *Cdx-2*^G^ variant, which has 70% reduced promoter activity (77); this variant correlated with an increased risk of MS in children who had ≤2 h/day of winter sun exposure (78). GWAS did not detect a *VDR* and MS association (61).

Some family studies have also detected linkage between *VDR* polymorphisms and T1D, but concerns about inconsistencies between populations and unknown functional impacts also apply here (79). Reasoning that a *VDR* and T1D association might only be evident if 25-OHD is sufficient to support calcitriol synthesis in cells relevant to T1D, investigators searched for this association as a function of latitude (79). They found a *VDR*^F^ and T1D association that varied in strength according to ambient winter UV light. These data emphasize the importance of analyzing genetic data in the context of environmental variables. GWAS did not detect a *VDR* and T1D association (62, 80).

Intriguing data suggest an epistatic interaction between *VDR* alleles and *HLA-DR* susceptibility loci in T1D as in MS. The *VDR*^F^–T1D association was only evident in patients who carried the high-risk HLA-DRB1*0301 allele (81). The HLA-DRB1*0301 allele like the HLA-DRB1*1501 allele harbors a putative VDRE in its promoter (70). Alleles without the putative VDRE were associated with disease resistance. These parallels suggest an influence of UV light and vitamin D on *HLA-DRB1* gene expression and presentation to CD4^+^ T lymphocytes of peptides relevant to T1D and MS etiology. The nature of the peptides and the timing and outcome of the presentation event are unknown, but could relate to thymic tolerance or peripheral T-cell responses to peptides from infectious agents. In any case, the positive findings regarding *VDR* polymorphisms provide genetic support for calcitriol and vitamin D receptor (VDR)-regulated transcriptional events as determinants of MS and T1D risk.

Additional evidence for vitamin D and calcitriol as sunlight’s signal transducers derives from vitamin D studies. An early study closely correlated childhood dental disease, serving as an accessible biomarker of exposure to low vitamin D status (82), with worldwide MS mortality (*r* = 0.78, *p* < 0.002) (83). The first vitamin D_3_ interventional study to inhibit autoimmune disease was performed in the murine experimental autoimmune encephalomyelitis (EAE) model of MS (84). Human case–control studies have correlated low vitamin D intake and low circulating 25-OHD with high MS risk (85–87). Importantly, MS risk correlated inversely with circulating 25-OHD independently of personal UV light exposure (88). Abundant data have now correlated circulating 25-OHD inversely with MS disease activity (30, 88–94). These data and the *CYP27B1* and *VDR* evidence contradict the view that UV light’s protective effects in demyelinating disease do not involve vitamin D (95).

In MS patients who had low vitamin D_3_ levels and were not taking disease-modifying drugs, supplementary vitamin D_3_ as a stand-alone intervention significantly reduced disease progression (96), and decreased new lesion formation and progression from optic neuritis to clinically definite MS (97). A vitamin D_2_ supplementation study did not report similar findings (98), but significant methodological flaws were noted in that study (99). Moreover, it is well known that vitamin D_2_ and vitamin D_3_ are not biologically comparable as regards their metabolism and ability to transcriptionally activate the VDR (100–102). Ongoing clinical studies are testing whether vitamin D_3_ as an add-on to disease-modifying drug therapy will improve drug efficacy (103). While this is a valid pharmaceutical question, it is separate from the question of whether vitamin D_3_ could be an etiology-based intervention to reduce the impact of autoimmune disease. Studies of vitamin D_3_ as a stand-alone intervention in individuals who have low vitamin D_3_ levels and who are not using disease-modifying drugs will be needed to address the etiological question. Such studies are needed and fully justified.

The T1D data also support vitamin D as sunlight’s protective signal transducer (6, 104, 105). The EURODIAB study correlated vitamin D_3_ supplementation in infancy with decreased T1D risk (odds ratio 0.67) (106), a finding that has been replicated (107). Finland, at 60–70°N, has the highest T1D incidence in the world (108). Finnish T1D incidence quadrupled as recommended vitamin D_3_ intakes for children decreased from 4000 to 400 IU/day between 1965 and 2005 (109). Another retrospective analysis found childhood vitamin D_3_ supplementation correlated with an 88% lower risk of T1D (110). These data imply that recommending higher vitamin D_3_ intakes for pregnant women, infants, and children might decrease T1D incidence by >75%. A very recent study demonstrated that life-long, high-dose vitamin D_3_ supplementation significantly reduced spontaneous diabetes in non-obese diabetic (NOD) mice (111). Finally, vitamin D_3_ interventions have improved glycemic control in T1D patients (112–114).

The association of UV irradiance and vitamin D with autoimmune disease risk is strong and consistent, shows a dose–response relationship, is temporally plausible and appears to be universal with respect to genotypes, dietary and smoking habits, and exposure to infectious and commensal organisms. Thus, it is reasonable to suggest that *vitamin D is probably the environmental factor with the greatest influence on the emergence of an autoimmune disease phenotype given a disease-susceptible genotype*. What remains to be done to satisfy the Bradford Hill criteria (115) is to rigorously test this relationship experimentally in humans, and to uncover plausible biological mechanisms that cohere with known facts of autoimmune disease. There is a growing consensus that a vitamin D_3_ interventional study for autoimmune disease prevention is needed and fully justified.

### T lymphocytes in autoimmune disease

The effort to decipher protective vitamin D mechanisms in autoimmunity focuses on T lymphocytes, because in failing to correctly discriminate between self and non-self, auto-reactive T cells drive target organ destruction (116). In animal models, auto-reactive T-cell transfer drives target organ destruction (117). The role of pathogenic auto-reactive T cells as autoimmune disease initiators unites these diseases under a common mechanistic umbrella.

Unequivocal evidence of T-lymphocyte involvement in human T1D came from transplantation studies. Transplanting pancreatic tissue from a healthy subject into his/her T1D-affected identical twin failed as a T1D therapy due to a T-cell-mediated attack on the transplanted pancreatic tissue unless T-cell immunosuppressive therapy was administered (118). Evidence for pathogenic CD4^+^ T lymphocytes in T1D is now very strong (10, 119).

Original evidence of T-lymphocyte involvement in MS pathogenesis came from studies showing T cells rapidly migrated from the periphery into the CNS of MS patients, where clonally restricted, activated T cells accumulated in nascent MS lesions (120). The T cells from individual MS patients showed dominant usage of specific T-cell receptor (TCR) alpha and beta chains and VDJ sequences, and specificity for myelin basic protein (MBP) peptides presented by HLA class II molecules (121). These observations argue strongly for involvement of HLA class II-restricted, auto-reactive CD4^+^ T cells in MS pathogenesis (122).

Newer experimental approaches have confirmed the pioneering studies. Analysis of the mRNA transcriptome in blood cells from MS patients pointed to antigen presentation, the immune synapse, and T-cell deregulation in MS pathogenesis (123, 124). GWAS identified genes related to CD4^+^ T-cell function in regions harboring putative susceptibility loci (61). Analysis of expression quantitative trait loci in autoimmune disease states (125) and genetic analysis of inter-individual variability in T helper (Th) activation as a function of ancestry and autoimmune disease susceptibility (126) also implicated CD4^+^ Th cell activation in autoimmune disease pathogenesis. In summary, consideration of gene–environment interactions, sunlight and vitamin D, and T lymphocytes as autoimmune disease initiators provides the rationale for investigating vitamin D mechanisms in CD4^+^ T cells in an effort to understand the molecular etiology of autoimmune disease.

## Vitamin D Metabolism from a T-Lymphocyte Perspective

### Calcitriol’s non-calcemic actions

The vitamin D system is an evolutionarily ancient and versatile system that coordinates a plethora of biological processes like cellular metabolism and growth, differentiation and death, organismal growth, reproduction, and immunity according to sunlight’s cues. The system’s signal transducing molecules are calcitriol, a small lipophilic hormone, and the VDR, a hormone-responsive transcriptional regulator (127). Calcitriol and mammalian VDR orthologs have functioned as sunlight sensors throughout >750 million years of evolution (128, 129). Many of the ancient organisms lacked calcified structures, so the vitamin D system must have originally supported non-calcemic functions (129). We focus on calcitriol’s actions in CD4^+^ T lymphocytes, but actions in other tissues, for example promotion of remyelination in the brain (130), insulin release from the pancreas (131), and intestinal barrier function in the colon (132), undoubtedly contribute to calcitriol’s potency as a chronic autoimmune and neurodegenerative disease inhibitor.

### Enzymes of vitamin D activation

Vitamin D metabolism has been well described elsewhere (127, 133–136). Cutaneous vitamin D_3_ generated by high energy UVB photons (290–315 nm) provides 90% of the human vitamin D requirement (108). Vitamin D_3_ synthesis varies seasonally at high latitudes, reaching a nadir 2 months after the winter solstice, and a zenith 2 months after the summer solstice (137). The biological half-life of 25-OHD_3_ is ~2 months, so this metabolite effectively integrates sunlight’s energy signal over time.

### At high latitudes, there is a period of “light starvation” when vitamin D synthesis is negligible

The higher the latitude, the greater is the period of light starvation. At 42°N, cutaneous vitamin D synthesis is negligible from November through February (138, 139). Vitamin D synthesis decreases with increasing skin pigmentation, sunscreen use, and advancing age. Vitamin D deficiency has rapidly become a worldwide health problem, due to life-style changes (indoor living and working; sunscreen use) that have reduced sunlight exposure (140, 141). Now, more than 75% of Caucasians and >90% of Blacks, Hispanics, and Asians in the USA have 25-OHD_3_ <75 nmol/L (30 ng/mL), double the number one decade ago (142). The rapid rise in vitamin D insufficiency correlates temporally with the rapid rise in MS and T1D incidence.

Two hydroxylation reactions produce the calcitriol from vitamin D_3_. The *CYP2R1*-encoded 25-hydroxylase converts vitamin D_3_ into 25-OHD_3_. The *CYP27B1*-encoded 1α-hydroxylase converts 25-OHD_3_ into calcitriol. Calcitriol induces *CYP24A1* gene encoding the 24-hydroxylase to convert calcitriol into inactive calcitroic acid. These hydroxylases are cytochrome P450 enzymes (133, 134). They are encoded in nuclear DNA, but the enzymes themselves localize to the mitochondrial membrane.

Many chronic diseases provisionally associated with vitamin D disproportionately impact individuals of African ancestry (AA). Lower serum 25-OHD in people of AA may be contributing to these health disparities (143). Serum 25-OHD levels are subject to genetic regulation (74), and polymorphisms in the *GC*, *DHCR7*, and *CYP2R1* genes have been correlated with circulating 25-OHD_3_ levels in people of European ancestry (EA) (144, 145). Motivated by the need to understand racial health disparities, recent research has investigated potential genetic contributions to circulating 25-OHD_3_ in AA and EA subjects. The AA subjects had different *GC* alleles encoding the vitamin D-binding protein (DBP) and lower circulating 25-OHD (146). In addition to binding vitamin D metabolites, the DBP functions in fatty acid transport, macrophage activation, and chemotaxis (147). A study of older male subjects from urban areas confirmed the association of *CYP2R1* variants with serum 25-OHD after correction for vitamin D intake, season of sampling, BMI, and other variables (148). This association was more robust in EA than AA subjects. The *GC* variants were associated with serum 25-OHD only in the EA subjects. Age, ancestry, vitamin D intake, and season of sampling explained 19 and 24% of the variance in AA and EA subjects, respectively. Adding genetic variants to the model explained an additional 1% (AA) and 4% (EA) of the variance. However, 72% (EA) and 80% (AA) of the variance was unexplained. Thus, genetic variants are very minor contributors to racial disparities in serum 25-OHD. The data also suggest some relevant parameters were either not considered or were subject to a large error in measurement. Unlike other reports, skin pigmentation was unrelated to serum 25-OHD in this study, and UVR exposure was marginally related to serum 25-OHD only in EA subjects. These data indicate serum 25-OHD derived primarily from vitamin D ingestion, which was assessed by a questionnaire. The questionnaire may have introduced error. Whether it assessed vitamin D supplement use was not stated. The authors previously reported that their EA subjects were more likely to use vitamin D supplements than the AA subjects (149). To generate insight into disparities in chronic diseases provisionally associated with vitamin D, it will be essential to discover dominant variables by integrating data on age, gender, life-style choices, skin color, UVR exposure, diet, vitamin D supplement use, and genetic variation with data on 25-OHD and disease phenotype.

### Paracrine calcitriol signaling to T cells

An important question from a CD4^+^ T-lymphocyte perspective is whether the calcitriol signal derives from the kidney (endocrine signaling) or from tissue-resident cells at sites of inflammation (paracrine signaling). Calcitriol has been classically viewed as an endocrine hormone, with renal synthesis and systemic distribution regulating mineral ion homeostasis and skeletal maintenance in target tissues. Blood calcitriol levels are maintained within very narrow limits and show minimal seasonal fluctuation (150), whereas many autoimmune diseases show fluctuating periods of relapse and remission. For example, MS disease activity fluctuates seasonally, correlating with and lagging changes in ambient UVB sunlight (31) and 25-OHD_3_ (29). These data are not consistent with endocrine signaling and instead support paracrine signaling to T lymphocytes.

Evidence that tissue-resident antigen-presenting cells (APC) produce calcitriol (151) and T cells express the VDR (152, 153) first suggested paracrine signaling between immune cells. Adding 25-OHD_3_ to cultures altered human T-cell responses only when APC were present to produce calcitriol (154). The APC required activation through pathogen-associated pattern recognition receptors, for example the toll-like receptors (TLR), and/or stimulation by cytokines, most notably interferon-gamma (IFN-γ), interleukin-2 (IL-2), and IL-15, to become calcitriol producers (136, 154–157).

Calcitriol synthesis has now been shown in many non-calcified mammalian tissues frequented by roving T lymphocytes, most notably the skin (158, 159), lung (160), colon (161, 162), brain (163), placenta, and other reproductive tissues (154, 157, 164), so paracrine signaling to T cells is a well-established concept. The *CYP27B1* transcripts are more abundant in tissues with barrier (skin, lung, colon) or reproductive function (maternal decidua, fetal trophoblast, testis) than in the kidney (158), supporting the thesis that biological protection at host–environment interfaces and environmental impacts on reproduction may have driven vitamin D system evolution. Available data suggest that *paracrine calcitriol signaling to T cells within tissues is likely the major pathway by which sunlight exerts its influence on the emergence of an autoimmune disease phenotype*.

It is valuable to consider how calcitriol signaling to CD4^+^ T lymphocytes could be compromised causing immune-mediated tissue damage. Research in the EAE model of MS demonstrated that insufficient vitamin D_3_ disrupted calcitriol synthesis in the CNS (84). Research in the NOD model of T1D demonstrated that reduced 1α-hydroxylase activity in APC disrupted signaling and contributed to diabetes (165). As detailed above, *CYP27B1* gene lesions compromise paracrine signaling. It is not widely appreciated that corticosteroids like prednisone (166), prednisolone (167), or dexamethasone (151) compromise paracrine signaling to CD4^+^ T cells because they inhibit calcitriol synthesis by activated innate immune cells *in vivo*. Corticosteroid inhibition of calcitriol synthesis in the airway may have contributed to negative results in the VIDA trial of vitamin D_3_ supplementation in asthma patients who were receiving concurrent corticosteroid therapy (168).

Deregulation of the *CYP24A1* gene promoter might also disrupt calcitriol signaling and promote a pro-inflammatory state. Epigenetic silencing of the *CYP24A1* promoter in placental tissue promoted calcitriol accumulation and an immune tolerant state at the maternal–fetal interface (169, 170). Expression of the *CYP24A1* gene differed between males and females. In the CNS of rodents with EAE, *Cyp24a1* gene expression was higher and calcitriol responsiveness lower in males than females (84, 171). Similarly in human T cells from MS patients and healthy controls, *CYP24A1* gene expression was higher and calcitriol responsiveness was lower in males than females (172). The rodent and human data suggest males may produce calcitriol at a rate equal to females but inactivate it faster. Gender differences in *CYP24A1* expression and vitamin D metabolism may underlie the stronger inverse correlation between ambient UV light and MS risk in women compared to men (173). Estradiol addition to male T cells decreased the *CYP24A1* transcripts and increased calcitriol responsiveness (172), suggesting estradiol may silence the *CYP24A1* gene to promote calcitriol accumulation (169, 170).

## Vitamin D Receptor Expression by CD4^+^ T Lymphocytes

### Vitamin D receptor and vitamin D-responsive elements

The VDR enables cells to respond to calcitriol. Early researchers demonstrated a protein with high affinity (*K*_d_ 0.1 nM) for calcitriol in activated human CD4^+^ T lymphocytes that was later identified as the VDR (152, 153). The VDR is a nuclear protein that dimerizes with the retinoid X receptor to regulate gene expression through VDRE in calcitriol-responsive genes (127). A VDRE is composed of two hexameric half-sites, arranged as direct repeats separated by three random base pairs, for example *GGTTCA*CGA*GGTTCA* (174, 175). Depending on the type of cell, the ligand-activated VDR–RXR complex recruits either coactivator complexes and cooperating transcriptional machinery or corepressor complexes to determine the nature of the transcriptional response from VDRE-containing target genes.

Antibody specificity problems have confounded investigations of VDR protein expression in T cells. All but one of the commercially available antibodies to the VDR bound non-specifically to cells and tissues from DeMay VDR-null mice (176, 177). These were confirmed by flow cytometry; all available antibodies either stained CD4^+^ T cells from DeMay VDR-null mice or did not yield a signal (171). The specificity problems have slowed progress in assessing VDR protein expression in T-cell subsets.

### VDR and CD4^+^ T-cell activation

Resting and activated T cells differ significantly in VDR expression; resting cells expressed fewer than 1000 VDR/cell and activation increased this number 10-fold (178). VDR expression peaked at 48 h post stimulation (179). In rodents, activated CD8^+^ T cells had higher VDR expression than CD4^+^ T cells (180).

Vitamin D receptor expression was needed for optimal human T-cell activation *in vitro* (181). Naïve T cells were VDR negative and responded weakly to TCR stimulation. Weak TCR signaling via the mitogen-activated protein kinase p38 pathway induced VDR expression. The T cells subsequently up-regulated phospholipase C-gamma1 (PLC-γ1) expression 75-fold, enabling them to flux calcium and become fully activated. PLC-γ1 induction appeared to be VDR-dependent. The dependence of T-cell activation on VDR expression has not been demonstrated *in vivo*.

### VDR and CD4^+^ T-cell subsets

Among rodent CD4^+^ T-cell subsets, IL-4-producing Th2 cells, IFN-γ-producing Th1 cells, and IL-17-producing Th17 cells all had abundant *Vdr* transcripts (182–185). Importantly, a source of estradiol in female mice (186), and a functional *Ifng* gene in male and female mice (171) were essential for Th1 and Th17 cell *Vdr* gene expression. Recent data on human Th1 and Th17 cells produced *in vitro* also show high *Vdr* gene expression (179).

Whether CD4^+^Foxp3^+^ Treg cells express the VDR is unclear. Very low *Vdr* transcript levels were observed in rodent CD4^+^Foxp3^+^ Treg cells produced *in vitro* (187) and rodent CD4^+^EGFP^+^ Treg cells generated *in vivo* and flow-sorted from the spleens of *Foxp3*^EGFP^ reporter mice (184). Those *in vivo* studies revealed an inverse relationship between *Foxp3* and *Vdr* gene expression, with many *Foxp3* transcripts and few *Vdr* transcripts in the Foxp3^+^EGFP^+^ T cells and the reverse pattern in the EGFP^−^ T cells. Consistent with low *Vdr* gene expression, calcitriol had no impact on flow-sorted CD4^+^Foxp3^+^EGFP^+^ T cells during activation *in vitro* (184). Calcitriol also had no impact on CD4^+^Foxp3^+^ T-cell proportions in the spleens of WT B6.*Cre*^−^*VDR*^fl/fl^ mice and B6.*Cre*^+^*VDR*^fl/fl^ mice with CD4^+^ T-cell-specific *Vdr* targeting, whether or not they were treated with calcitriol (184). These data suggest fully differentiated, mature rodent CD4^+^Foxp3^+^ T cells may not be calcitriol responsive. Contrary to this view, VDR-dependent down-regulation (187) and up-regulation (185) of *Foxp3* transcription in rodent CD4^+^Foxp3^+^ T cells have both been reported. It will be interesting to learn the molecular details of *Vdr* and *Foxp3* gene expression control as research in this area progresses.

### CD4^+^ T cells are calcitriol targets in autoimmune disease

Selective *Vdr* gene inactivation experiments in rodents provided unequivocal evidence that calcitriol targets CD4^+^ T lymphocytes for the purpose of immune system regulation (184). Reciprocal mixed bone marrow chimera studies established that hematopoietic cell *Vdr* gene expression was necessary for calcitriol to inhibit EAE induction. In fact, chimeric mice lacking the VDR in hematopoietic cells had a particularly aggressive EAE disease course. Subsequently, mice with CD4^+^ T-cell-specific *Vdr* targeting were constructed. Evaluation of T-cell subsets in the periphery of naïve T-cell *Vdr*-targeted mice assured that this genetic manipulation did not influence T-cell proportions during thymic selection. Neither vitamin D_3_ nor calcitriol inhibited EAE induction in mice with CD4^+^ T-cell-specific *Vdr* targeting (184). Thus, EAE data establish that calcitriol exerts protective biological effects against autoimmunity *in vivo* through the nuclear VDR in CD4^+^ T lymphocytes. These data do not rule out vitamin D effects on myeloid cells for a pathogen protective immune response (188). Studies in mice with CD4^+^ T-cell-specific *Vdr* targeting (184), and in humans with loss-of-function mutations in the *VDR* gene (189) support the view that *the outcome of calcitriol signaling within CD4*^+^ *T cells is likely a major determinant of sunlight’s influence on the emergence of an autoimmune disease phenotype*.

## Vitamin D and Thymocyte Selection

### Thymic negative selection in autoimmune disease

The TCR repertoire is shaped in the mammalian postnatal thymus, and autoimmune disease risk is believed to reflect in part defects in this process (190). When immature CD4^+^CD8^+^ thymocytes engage cortical thymic epithelial cells expressing self peptides embedded in major histocompatibility complex (MHC) molecules, those that bind self MHC molecules with adequate affinity undergo positive selection and develop into CD4^+^ or CD8^+^ T cells. Those that bind with inadequate affinity for survival signaling undergo apoptosis. Surviving thymocytes migrate to the thymic medulla where they engage medullary thymic epithelial cells (mTEC) presenting self peptides, including peptides derived from tissue-restricted proteins. Cells that bind self peptides with high affinity receive apoptotic signals. The survivors are released to form the pre-immune TCR repertoire.

An analysis of human autoimmune TCR–peptide–MHC complexes revealed structural anomalies compared to anti-microbial TCR–peptide–MHC complexes suggesting these TCR may have escaped thymic negative selection (191). For example, a TCR from an MS patient recognized both a DRB1*1501-restricted MBP peptide and a DRB5*0101-restricted EBV peptide, suggesting this TCR may have escaped negative selection become activated by a pathogen (192). A negative selection failure during early life is believed to release potentially pathogenic T cells that initiate autoimmune disease when a cross-reactive pathogen peptide activates them (116).

### Vitamin D and thymic negative selection

Sunlight and vitamin D_3_ exert their strongest influence on MS risk during childhood, which coincides with the peak period for T-cell selection in the postnatal thymus (19). Intriguingly, recent data quantifying signal joint TCR excision circles (sjTRECs) in human cord blood T cells as a function of season demonstrated seasonal variability in thymic output (193). May-born infants had significantly lower circulating 25-OHD_3_ and higher sjTRECs/10^5^ T cells than November-born infants. The sjTRECs are extra-chromosomal DNA circles formed during TCR gene rearrangement. They are not replicated as mature peripheral T cells divide, so they serve as markers of recent thymic emigrants in the peripheral T-cell repertoire. The data suggest a positive influence of 25-OHD_3_ on thymic negative selection. Rigorous investigation of this possibility is warranted because of the significance such an influence would have on autoimmune disease risk acquisition, and the insight it would provide into the timing of risk acquisition and therefore the timing of etiology-based intervention strategies to prevent disease.

## Vitamin D and CD4^+^ Th1 and Th17 T Cells

### Effector T-cell anti-microbial response, cross-reactivity, and autoimmune disease

Exposure to common infectious or commensal organisms triggers activation of those CD4^+^ T cells capable of recognizing foreign antigens in an MHCII context. The biological imperative to provide adequate immune cover for the host inevitably results in some effector T-cell cross-reactivity between foreign and self peptides due to molecular mimicry (116). Peptide antigen diversity is orders of magnitude larger than TCR diversity (194), and TCR–peptide–MHC binding shows conformational plasticity, relatively low affinity, and rapid off-kinetics (191, 195). The immune system has evolved mechanisms to prevent overly aggressive T-cell-mediated responses that cross-react with host peptides from damaging host tissue (196). These mechanisms include inhibition of pro-inflammatory cytokine synthesis, and Treg cell-mediated restriction of T-cell expansion through inhibitory receptors like cytotoxic T-lymphocyte antigen 4 (CTLA4), induction of T-cell anergy, and cell death signaling (197). Intriguing new research has documented a central role for the vitamin D system in the development of Treg cells and the termination of effector T-cell responses.

### Modeling human autoimmune disease

Two animal models have been particularly useful in developing mechanistic knowledge of direct calcitriol actions in CD4^+^ T cells, murine EAE (198, 199), and the NOD mouse (200). These animal models show strong parallels to their respective human diseases, especially in the immunological aspects of disease. The parallels are imperfect, for example regarding the role of human microbial exposures, because our understanding of human autoimmunity is incomplete. Nevertheless, hypothesis testing is more facile in animal models where direct manipulation of contributing factors is possible. Calcitriol inhibited autoimmune disease in the EAE and NOD models (201–203). Working back and forth between animal models and human disease has allowed rapid forward progress to be made in understanding how the vitamin D system modulates immunity.

### Vitamin D and Th1 cells

Disease prevention studies in animal models have suggested that calcitriol directly inhibits encephalitogenic Th1 cells. Calcitriol inhibited EAE induction (182, 183, 185, 202–205), and targeting the *Vdr* gene specifically in CD4^+^ T cells abrogated this inhibition (184). However, calcitriol had no effect on CD4^+^ T-cell priming in the periphery, myelin-specific T-cell trafficking into the CNS, or IFN-γ synthesis by freshly explanted, purified CD4^+^ T cells from EAE mice (182). Instead, the CNS-infiltrating myelin-specific CD4^+^ T cells displayed an anergic phenotype in the calcitriol-treated mice. These findings have been confirmed (171, 184). In another strain, calcitriol inhibited EAE induction, but the mechanism reported was a decreased Th1 cell frequency in peripheral lymphoid tissues (206).

The mechanisms involved in amelioration of established autoimmune disease may differ from those involved in autoimmune disease prevention. Administering calcitriol to rodents with established EAE resulted in very rapid disease remission that correlated with fewer CNS CD4^+^ T cells, loss of IFN-γ-production, and a significant decrease in CNS pathology (207–210). Induction of Treg cells in the EAE studies is discussed below.

A short course of oral calcitriol treatment in healthy male volunteers had no measurable effect on circulating IFN-γ (211). Likewise, in two studies carried out in MS patients, vitamin D_3_ supplementation had no measurable effect on circulating IFN-γ (96, 212). In a third study performed in healthy volunteers, vitamin D_3_ supplementation decreased the mean percentage of Th1 cells in the circulation from 20 to 17% (213).

### Vitamin D and Th17 cells

Disease prevention studies in animal models have also suggested that calcitriol directly inhibits encephalitogenic Th17 cells. Experimental autoimmune uveitis (EAU) induced by immunization of mice with a retinal antigen serves as model for human autoimmune uveitis. Oral calcitriol prevented as well as partly reversed EAU disease (214). In this model, calcitriol treatment *in vivo* of mice impaired T-cell commitment to the Th17 lineage as well as Th17 production of IL-17.

**Figure 1:**
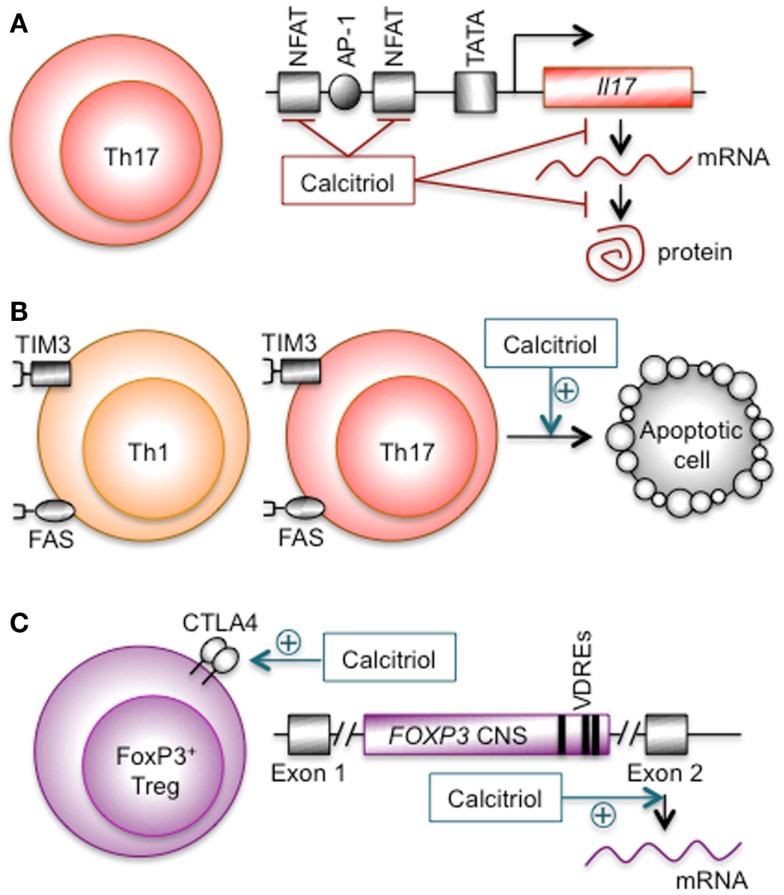
**Proposed calcitriol mechanisms in effector Th1, Th17, and FoxP3^+^ Treg cells**. **(A)** Calcitriol and IL-17 synthesis in Th17 cells. Calcitriol inhibited IL-17 synthesis in a VDR-dependent manner by a post-transcriptional mechanism (183, 187). It also blocked NFAT, recruited histone deacetylase, and sequestered Runx1 to suppress murine *Il17* gene transcription (185). **(B)** Calcitriol and effector CD4^+^ T-cell apoptosis. Calcitriol increased CD4^+^ T-cell sensitivity to extrinsic cell death signals that may have been transduced through the FAS ligand–FAS–caspase-8 and/or the galectin-9–TIM3–calpain pathways (208–210, 216). **(C)** Calcitriol and *FOXP3* gene transcription. Calcitriol increased the transcription of the murine *FoxP3* (185) and human *FOXP3* genes (217). Three VDREs that functioned as calcitriol-dependent enhancers were identified in a conserved non-coding sequence (abbreviated CNS in this diagram) between exons 1 and 2. Calcitriol also increased CTLA4 protein expression (218–220).

Similar results were reported in the EAE model. The spleens of calcitriol-treated mice had fewer splenic Th17 cells and lower IL-17 production than the placebo controls (183, 187). Mice with global inactivation of the *Vdr* gene had activated Th17 cells that overproduced the cytokine (215). Calcitriol treatment reduced Th17 cells in the CNS in an EAE prevention study (171) and in two EAE treatment studies (185, 216). Different mechanisms were suggested to explain these findings (Figure 1A). In one study, calcitriol did not suppress *Il17* gene transcription, but inhibited Th17 cell IL-17F production in a VDR-dependent manner by a post-transcriptional mechanism (183). In another study, calcitriol suppressed *Il17* gene transcription by blocking nuclear factor for activated T cells (NFAT), recruiting histone deacetylase, and sequestering Runt-related transcription factor 1 (Runx1) (185). In the third study, elimination of Th17 cells by a programed cell death mechanism was suggested (216). The reconciliation of these divergent mechanisms awaits further investigation.

Studies of calcitriol and Th17 cell activity in humans are at an early stage. Cell culture studies have shown that adding calcitriol to activated CD4^+^ T cells reduced the frequency of Th17 cells (218, 221), an effect that was more pronounced in cultures of T cells from women than from men (172). New data have extended these observations in a vitamin D_3_ dose escalation study performed in healthy controls during the UV-restricted winter months (213). As supplementary vitamin D_3_ increased from 2000 to 8000 IU/day, the mean serum 25-OHD rose from 30 ± 12 to 159 ± 29 nmol/L, and the proportions of Th1 and Th17 cells in the peripheral blood decreased. There appeared to be a threshold effect with at least 70 nmol/L of 25-OHD triggering a mean 40% decrease in circulating Th17 cells in the healthy controls. Other studies found no correlation between Th17 cells and 25-OHD in MS patients (222), and no effect of supplementary vitamin D_3_ on Th17 cells in MS patients (223). Why the human data are conflicting is not clear, but vitamin D_3_ status at enrollment, vitamin D_3_ dose, dose frequency (135), use of disease-modifying drugs, and timing of sampling relative to estrogen cycling in women are potential confounding factors. Nevertheless, given the strong animal modeling data and some human *in vivo* data that are consistent with animal modeling, it is reasonable to suggest that *vitamin D may significantly influence the emergence of an autoimmune disease phenotype by dampening pathogenic Th17 cells and IL-17 synthesis*.

### Vitamin D and Th2 cells

Analyses of Th2 cells *in vivo* have also yielded inconsistent results. Administering calcitriol to mice before EAE induction increased the IL-4 transcripts in the lymph nodes and in the CNS compared to the placebo controls (204). Also, targeted disruption of the *IL-4* gene moderately decreased the protective function of calcitriol in EAE (224). A subsequent report found no significant differences between calcitriol-pretreated and placebo-pretreated B10.PL mice with regard to IL-4 mRNA in the lymph nodes or the CNS after immunization with MBP (182). There was also no effect of calcitriol on the IL-4 protein synthesis per Th2 cell. Another report found administering calcitriol to Biozzi AB/H mice before EAE induction had no effect on the IL-4-producing Th2 cell frequency (206). Thus, there are some inconsistencies regarding IL-4 that remain to be resolved. Since calcitriol inhibition of EAE decreased slightly in *IL-4*-null mice, there may be some IL-4 contribution to the mechanism (224), but it is possible that Th2 cells were not the source of the protective IL-4.

The IL-5-producing Th2 cells have a pathogenic role in human asthma. When CD4^+^ T cells from asthma patients were stimulated with dust mite allergen in the presence of calcitriol, the hormone decreased IL-5, IL-9, and IL-13 production (225). Calcitriol’s actions to promote IL-10-producing Treg cells in asthma patients are discussed below.

## Vitamin D and CD4^+^ T-Cell Programed Cell Death

### Effector T-cell apoptosis and autoimmune-mediated tissue damage

When effector CD4^+^ T cells are no longer needed for anti-microbial defense, programed cell death mechanisms remove them to limit immune-mediated tissue damage. Several mechanisms used by CD4^+^ Treg cells (197, 226), astrocytes and neurons (227, 228), and a few other types of cells to control effector CD4^+^ T cells involve apoptosis induction. In individuals predisposed to autoimmunity, it appears that effector CD4^+^ T cells resist cell death mechanisms, proliferate, and continue to produce inflammatory molecules that damage the host’s tissues.

The effector T lymphocytes from the T1D-susceptible NOD mice were resistant to cell death signals. The resistance to cell death mapped to a genomic region encompassing the *Ctla4* gene, which was defectively expressed on T cells from these mice (229). In human autoimmunity, myelin-specific CD4^+^ T cells from MS patients provide an example of defective cell death mechanisms (230). These T cells were defective in the FAS–caspase 8 cell death pathway (231) and the galectin-9–TIM-3 cell death pathway (232). It was intriguing that myelin-specific T cells from MS patients with benign disease had a significantly augmented galectin-9–TIM-3 cell death pathway compared to MS patients with active disease (Saresella et al., ECTRIMS 2011, Abstract P324). Moreover, MS disease activity fluctuated seasonally, with a high frequency of new lesions following a period of reduced ambient UV light and *vice versa* (31). Taken together, the seasonal fluctuations in MS disease activity and disease activity-associated fluctuations in T-cell apoptosis resistance hint at a possible causal relationship between ambient UVB light, vitamin D_3_ supplies, myelin-specific T-cell susceptibility to cell death, and demyelinating disease activity.

### Vitamin D regulation of effector T-cell apoptosis

A causal relationship between calcitriol, myelin-specific T-cell responsiveness to cell death signals, and demyelinating disease activity has been demonstrated in the animal model of MS (Figure 1B). In animals with EAE, calcitriol treatment *in vivo* increased the susceptibility of pathogenic myelin-specific CD4^+^ T cells to extrinsic, CNS-derived apoptotic signals (207). This treatment induced the pro-apoptotic gene encoding caspase-8-associated protein, which is essential for FAS-mediated apoptosis, and repressed cellular inhibitor of apoptosis protein 2 (cIAP-2), an apoptosis inhibitor (209). Effector T-cell death is triggered in the CNS by astrocyte- and neuron-mediated activation of the T-cell FAS death pathway (227, 228, 233–235). In the calcitriol treatment study, apoptotic CD4^+^ T cells were evident in CNS lesions and IFN-γ production ceased by 12 h post treatment; within 1 day, the CD4^+^ T-cell numbers were 60% reduced, correlating with abatement of clinical disease signs by day 3 (208, 210). These T-cell changes were not observed in the periphery or *in vitro*. A transient increase in Helios^+^FoxP3^+^ Treg cells coincided with the loss of effector T cells suggesting the Treg cells may have played an active role in effector T-cell elimination (216). In the NOD model of T1D, peripheral T cells were resistant to programed cell death signals, and calcitriol treatment restored their sensitivity to these signals (229, 236–238). These results support a direct calcitriol action on effector T cells to promote sensitivity to apoptotic signals.

Vitamin D may also influence other cell death pathways in effector CD4^+^ T cells. Treg cells expressing galectins can trigger effector T-cell death via the TIM3 pathway (226). The N-linked glycans, in particular terminal galactosyl residues, are essential for galectin-9 binding to TIM3 and induction of cell death through calpain and caspase-1 activation (239, 240). Importantly, calcitriol enhanced α-mannosidase *N*-acetyl-glucosaminyl transferase 1, the rate-limiting enzyme in N-linked glycan synthesis (241), UDP-galactose: beta *N*-acetyl glucosamine-beta-1,3-galactosyl transferase, and calpain (209). Thus, calcitriol may improve T-cell sensitivity to the galectin-9–TIM3–calpain cell death pathway, which was impaired in MS patients (232). Together, the animal modeling and human *in vivo* data linking vitamin D and calcitriol with effector T-cell apoptosis suggest that *vitamin D may significantly influence the emergence of an autoimmune disease phenotype by increasing effector CD4^+^ T-cell sensitivity to extrinsic cell death signals*.

## Vitamin D and CD4^+^ T-Regulatory Lymphocytes

### Treg lymphocytes in autoimmune disease

CD4^+^ Treg cells are defined functionally by their ability to limit prolonged effector T-cell activation, thereby preventing autoimmune-mediated pathology (242). T lymphocytes that suppress autoimmunity were first demonstrated in mice (243–246). Subsequently, their existence was confirmed in humans (247). Several types of Treg cells exist differing in origin, phenotype, and function. The most intensely studied are those for which FoxP3 serves as the definitive transcription factor in lineage specification (248). One FoxP3^+^CD4^+^ Treg cell subset arises during thymic development (tTreg), whereas another arises during TCR engagement in the periphery (pTreg) (249). These FoxP3^+^CD4^+^ Treg cell subsets are non-functional in male *scurfy* mice and boys with the multi-organ autoimmune disease immune-dysregulation polyendocrinopathy enteropathy X-linked syndrome (IPEX), due to loss-of-function mutations in the X-linked rodent *Foxp3* and human *FOXP3* genes, respectively. The FoxP3 protein serves as a biomarker for this Treg lineage.

The T-regulatory cell type 1 (Tr1) cells differ from FoxP3^+^CD4^+^ Treg cells in origin, phenotype, and function (250). The CD4^+^ Tr1 cells are peripheral memory T lymphocytes that are anergic and do not express FoxP3 (251). Selective biomarkers for Tr1 cells in humans and rodents are co-expression of integrin alpha 2 subunit (CD49b, a cell adhesion molecule) and lymphocyte activation gene 3 (LAG-3 or CD223, a negative regulator of effector T-cell function) (252). Upon CD3 stimulation and co-stimulation via the complement regulator CD46 in the presence of IL-2, the CD4^+^ Tr1 cells rapidly proliferate and secrete large amounts of IL-10 and TGF-β to exert suppressive function (253). The Tr1 cells also release granzyme B to specifically lyse APC of myeloid origin, effectively terminating further effector T-cell activation.

Functional defects in FoxP3^+^CD4^+^ Treg cells have been described in multiple autoimmune diseases (254). Such defects were first reported and confirmed in peripheral blood T cells from MS patients (255, 256). Additional evidence emerged for reduced CD4^+^ Treg cell generation in the thymus of MS patients (257, 258). The impairment correlated with reduced FoxP3 expression in MS patient T cells (259, 260). Studies of FoxP3^+^CD4^+^ Treg cell defects in MS have been reviewed (261, 262). Functional defects in FoxP3^+^CD4^+^ Treg cells have also been extensively described in T1D (263). Below, we review research suggesting calcitriol enhances the expression of Helios, a positive regulator of FoxP3, and of FoxP3 itself.

Functional defects in IL-10-producing CD4^+^ Tr1 cells have also been described in many autoimmune diseases (250). An early report demonstrated that T cells from T1D patients showed extreme polarization toward a pro-inflammatory Th1 phenotype, whereas the T cells from the non-diabetic, HLA-matched control subjects showed an extreme bias toward the IL-10-secreting Treg phenotype (264). Functional defects in Tr1 cells have also been extensively studied in MS. Stimulation of MS patient T cells *in vitro* via CD3 and CD46 elicited very little IL-10 production compared to controls (265). These findings have been confirmed (261, 266, 267) and extended to a primate model of MS (268). Functional defects in IL-10-producing CD4^+^ Tr1 cells have also been described in asthma and rheumatoid arthritis (269). Below, we review research suggesting calcitriol enhances the expression of IL-10 and controls the Th1–Tr1 switch through modulation of CD46, a positive regulator of IL-10.

### Cutaneous UVB light exposure and Treg lymphocytes

Cutaneous UVB light exposure and *in situ* vitamin D metabolism may have a particularly important role in cutaneous Treg cell development. Cutaneous UVB exposure promotes T-cell-mediated immunity to microbial pathogens; Finsen received the 1903 Nobel Prize in Medicine for phototherapy of skin diseases (270). More recently, the role of cutaneous UVB exposure in peripheral immune tolerance has been recognized (271). The dual role of UVB exposure illustrates how the vitamin D system may have evolved to promote anti-pathogen responses at environmental interfaces, and subsequently to terminate these responses before the tissues sustain immune-mediated damage.

Ground-breaking animal modeling studies revealed how cutaneous UVB exposure promotes peripheral immune tolerance at the molecular level (272). Infection increased keratinocyte expression of receptor-activator of NF-kappaB ligand (RANKL). In transgenic mice with keratinocytes overexpressing RANKL, the keratinocytes signaled receptor-activator of NF-kappaB (RANK)-expressing Langerhans cells to induce IL-10-producing pTreg cells in the skin-draining lymph nodes. These pTreg cells suppressed the contact hypersensitivity response to cutaneous antigens in the original host animal and in adoptive transfer host animals. Furthermore, when skin was grafted from WT mice or *Tnfrsf11a*-null mice lacking RANKL onto WT host animals, UVB irradiation of the WT but not the *Tnfrsf11a*-null skin graft prevented graft rejection. These experiments established that keratinocyte RANKL expression was necessary for UVB exposure to induce IL-10-producing pTreg cells specific for the grafted skin to maintain peripheral tolerance.

Importantly, cutaneous UVB exposure promoted pTreg cell development by a calcitriol and VDR-dependent mechanism (273). Keratinocytes have a complete vitamin D_3_ biosynthetic pathway which when stimulated by infection, injury, or light produced 2–5 nmol/L of calcitriol *in situ* (159, 274), about 20- to 50-fold higher than calcitriol in the blood plasma of non-pregnant women (275). Furthermore, calcitriol transcriptionally activated the murine (*Tnfrsf11a*) and human (*TNFRSF11A)* genes encoding RANKL through highly evolutionarily conserved and functionally active VDREs in the promoter regions, as described in osteoblasts (276–278), murine and human T lymphocytes (279, 280), and keratinocytes (127). In the animal model of cutaneous hypersensitivity, topically applied calcitriol mimicked cutaneous UVB exposure by inducing keratinocyte RANKL expression and stimulating IL-10-producing pTreg cells by a mechanism that was VDR-dependent (281, 282). Cell labeling studies demonstrated that the pTregs trafficked between the skin, the skin-draining lymph nodes, and the circulation (283). Calcitriol suppressed T-cell expression of the gut-homing receptors and increased T-cell expression of CC chemokine receptor 10, enabling T-cell migration to the skin-specific chemokine CCL27 produced by keratinocytes (100). In summary, seminal studies of experimental UVB-induced peripheral tolerance in rodents established that a calcitriol- and VDR-dependent pathway exists involving (i) keratinocyte synthesis of calcitriol and stimulation of RANKL expression, (ii) signaling to RANK-expressing Langerhans cells, (iii) movement of the Langerhans cells to the skin-draining lymph nodes, (iv) induction of cutaneous antigen-specific, IL-10-producing pTreg cells, and finally (v) movement of the pTregs to the skin to prevent cutaneous antigen-specific effector T cells from degrading the epidermal barrier.

New research has begun to translate this knowledge to humans. Subjects from the North of Scotland who had skin disease were recruited between December and March for a phototherapy study (284). The subjects had a mean baseline 25-OHD level of 34 nmol/L and 0.5% FoxP3^+^ pTreg cells as a percentage of blood CD3^+^ T cells. After 4 weeks of phototherapy, the mean 25-OHD was 78 nmol/L and the FoxP3^+^ pTreg cell percentage was 1.6%. Another study demonstrated that UVB exposure substantially expanded pTreg cell numbers in the skin, and these skin pTreg cells appeared to have derived from peripheral immune organs (285). The studies in animals and humans implicating calcitriol as a positive regulator of the *Tnfsf11a* and *Tnfrsf11a* genes and cutaneous FoxP3^+^ pTreg cell induction suggest that *during periods of light starvation, phototherapy may be a particularly efficient method of influencing the emergence of an autoimmune disease phenotype by increasing CD4^+^CD25^+^FoxP3^+^ Treg cell development and suppressive function*.

### Vitamin D and the IL-10–IL-10R pathway

Interleukin-10 is a particularly important anti-inflammatory cytokine that protects the CNS (286), the airway (287), the gastrointestinal tract (288), and other tissues from immune-mediated pathology (289, 290). Disruption of the IL-10–IL-10R signaling pathway results in severe inflammatory disease (291).

Early research in two rodent models suggested that calcitriol promotes the development of IL-10-producing Treg cells. In the EAE model, suppressor T cells inhibited disease by an IL-10-dependent mechanism (292, 293). Calcitriol inhibited EAE not by blocking T-cell priming in the periphery or effector T-cell trafficking into the CNS, but instead by inducing *Rag-1* gene-dependent regulatory lymphocytes (182). Subsequent work demonstrated that activation of human and mouse T cells in the presence of dexamethasone and calcitriol *in vitro* yielded IL-10-producing Tr1 cells (294). Furthermore, vitamin D_3_ or calcitriol-mediated inhibition of EAE required functional *Il10* and *Il10R* genes, bidirectional IL-10–IL-10R signaling between hematopoietic and non-hematopoietic cells (205), and a functional *Vdr* gene in CD4^+^ T cells (184). Calcitriol had no effect on IL-10R expression (205). In the NOD model of T1D, calcitriol increased the frequency of Treg cells in the pancreatic lymph node (295), and global *Vdr* gene inactivation decreased the frequency of these cells (296). A positive calcitriol influence on T-cell IL-10 synthesis was observed *in vitro* (297). These animal studies suggested calcitriol may inhibit autoimmune disease at least in part through a VDR-dependent action on CD4^+^ T cells to induce IL-10-producing Treg cells.

There is limited information on a possible direct link between vitamin D and IL-10 in humans. An early study found a seasonal variation in the cord blood IL-10 level that correlated directly with the 25-OHD for infants born at 51°N (298). The 25-OHD levels were 99% higher and IL-10 was 43% higher in samples obtained in the summer compared to samples obtained in the winter. Investigations of calcitriol effects on IL-10 synthesis by human T cells activated *in vitro* have yielded conflicting results with reports of both enhancement (172, 221) and inhibition (179). A very interesting recent study examined the effect of adding 25-OHD_3_ to cultures of lymphocytes from healthy controls and from hereditary vitamin D-dependent rickets (HVDRR) patients with loss-of-function mutations in the *VDR* gene (189). The 25-OHD_3_ increased IL-4, IL-10, and IFN-γ production from control lymphocytes, but not patient lymphocytes, demonstrating calcitriol synthesis and VDR dependence of IL-10 induction *in vitro*.

### Vitamin D and Treg cells in asthma

Asthma research has yielded important new insights regarding vitamin D and Treg cells (299). One of them relates to the balance between immunity to pathogens and self-tolerance. Calcitriol ingestion by healthy volunteers and calcitriol addition to cultures increased CD4^+^ T-cell IL-10 secretion and TLR9 expression (300). Adding TLR9 agonists to the cultures decreased T-cell IL-10 production and suppressive function. These findings are significant because they suggest pathogen signaling through TLR9 could suspend T-cell suppressive function temporarily until an infection has been cleared. A second insight relates to calcitriol enhancement of T-cell surface molecules that dampen immune responses. In this case, calcitriol up-regulated CD200 on human peripheral and respiratory tract CD4^+^ T cells *in vitro*, and there was a trend toward up-regulation *in vivo* in healthy, but not asthmatic individuals (301). CD200 is an immunoglobulin superfamily member; it imparts a unidirectional negative signal to suppress innate and adaptive immune responses.

Yet another insight from the asthma research relates to the distinction between CD4^+^FoxP3^−^IL-10^+^ Tr1 cells and CD4^+^FoxP3^+^IL-10^−^ Treg cells. Systemic vitamin D status correlated directly with airway levels of IL-10 and CD4^+^FoxP3^+^ T cells in pediatric asthma patients and in healthy controls (302, 303). In cultures of human peripheral blood T cells, adding calcitriol at moderate levels (10^−8^ mol/L) increased the frequency of CD4^+^IL-10^+^ Tr1 cells, whereas higher calcitriol (10^−6^ mol/L) increased the frequency of CD4^+^FoxP3^+^ T cells. However, there was little co-expression of FoxP3 and IL-10, and IL-10 impaired calcitriol-mediated enhancement of FoxP3. Modulating the cytokine environment to include TGF-β together with calcitriol favored CD4^+^FoxP3^+^ T-cell outgrowth (304). The CD4^+^IL-10^+^ and CD4^+^FoxP3^+^ T-cell populations had equivalent suppressive activity *in vitro*, although their suppressive mechanisms were IL-10-dependent and IL-10-independent, respectively.

These data are highly significant for two reasons. First, they demonstrate that the vitamin D system supports two phenotypically and functionally distinct Treg cell populations. Secondly, they suggest that as infections are resolved, calcitriol-supported development of CD4^+^FoxP3^−^IL-10^+^ Tr1 cells and CD4^+^FoxP3^+^IL-10^−^Treg cells may proceed in an ordered sequence to perform slightly different functions, for example attenuating innate and adaptive immune responses by IL-10-dependent and IL-10-independent mechanisms, respectively. Many interesting questions remain regarding the underlying mechanisms of Treg cell immune response regulation in the airway, and the generality of these findings to other interfaces between the environment and the host organism where immunity to pathogens must be balanced with self-tolerance.

### Vitamin D and FoxP3^+^ Treg lymphocytes

Above, we summarized early animal modeling data suggesting calcitriol promoted the development of IL-10-producing Treg cells before FoxP3 expression was in use as a Treg biomarker. New animal modeling data suggest calcitriol may be a positive regulator of the murine *FoxP3* gene itself (185), and of the *Ikzf2* gene encoding Helios (216), a transcription factor that binds to the *FoxP3* promoter and stimulates its transcription (305–307).

CD4^+^Foxp3^+^ Treg cell studies in humans are scarce and conflicting. Two reports correlated 25-OHD with circulating CD4^+^Foxp3^+^ Treg cell percentages (308, 309). One study correlated the suppressive capacity of the CD25^+^CD4^+^ Treg cells with serum 25-OHD levels in MS patients (222), but this association could not be substantiated upon vitamin D_3_ supplementation (223). A more recent study correlated seasonal increases in 25-OHD and calcitriol in healthy men with increased Treg cell Foxp3 expression, a drop in memory CD4^+^CD45RO^+^ T cells, a reduced capacity for T-cell pro-inflammatory cytokine production, and increased CCR4, CCR6, CLA, CCR9, and CCR7 levels for homing to skin, gut, and lymphoid tissue (310). Seemingly contradictory results were reported for infants whose 25-OHD levels and T-cell subsets in cord blood showed no correlations (311). Potential confounders in human studies are differences in phenotypic markers used in the analyses, vitamin D_3_ status when beginning vitamin D_3_ supplementation, supplementation protocols (135), use of disease-modifying drugs in patients, and timing of sampling relative to estrogen cycling in women.

Examining human T cells in culture documented an influence of calcitriol on FoxP3 (Figure 1C). Activation of human CD4^+^CD25^−^ T cells *in vitro* in the presence of calcitriol resulted in greater numbers of CD4^+^CD25^+^FoxP3^+^ Treg cells (221), particularly for female T cells (172). Addition of calcitriol to human CD4^+^CD25^−^ T-cell cultures increased CTLA4 (CD152) and FoxP3 protein expression, the latter requiring the presence of IL-2 (218). The increase in CTLA4 and FoxP3 protein expression was confirmed for MS patient T cells (219), and the CTLA4 increase was confirmed for T1D patient T cells (220). The suppressive function of the calcitriol-induced CD4^+^CD25^+^FoxP3^+^ Treg cells was confirmed in many reports (218, 220, 221, 312).

Calcitriol positive regulation of CTLA4 is critically important, because this protein is an essential negative regulator of immune responses (313, 314). Humans who were heterozygous for loss-of-function mutations in the *Ctla4* gene developed a highly penetrant autoimmune syndrome with strong similarity to IPEX. Specifically, they displayed loss of CD4^+^FoxP3^+^ Treg cell suppressive function, hyperactivation of effector T cells, lymphocyte infiltration of multiple target organs, and fatal autoimmune disease. CTLA4 strips CD80 and CD86 from the APC by endocytosing and degrading these proteins to block their co-stimulation of CD28 on T cells (315). Calcitriol enhancement of this mechanism may account in part for the hormone’s actions as an immune response terminator.

Intriguing new data complemented the rodent data (185) and revealed that calcitriol increased human *FOXP3* gene expression by a transcriptional mechanism (217). The human *FOXP3* and murine *Foxp3* genes share homology in a conserved non-coding sequence (+1714 to +2554 relative to the *FOXP3* transcriptional start site) that functions as an enhancer (316). Within this enhancer region, investigators identified three VDREs that enhanced promoter activity in a calcitriol-dependent manner (217). Moreover, T-cell stimulation through CD3 and CD28 in the presence of IL-2 and calcitriol generated CD4^+^CD25^+^FoxP3^+^ Treg cells that inhibited target CD4^+^ T-cell proliferation by a cell contact- and FOXP3-dependent mechanism. Collectively, the data demonstrating calcitriol enhancement of *Ikzf2*, *FOXP3*, and *Ctla4* gene expression suggest that *vitamin D may significantly influence the emergence of an autoimmune disease phenotype by increasing CD4^+^CD25^+^FoxP3^+^ Treg cells and/or their suppressive function*.

### Vitamin D, CD46, and IL-10-producing Tr1 lymphocytes

**Figure 2:**
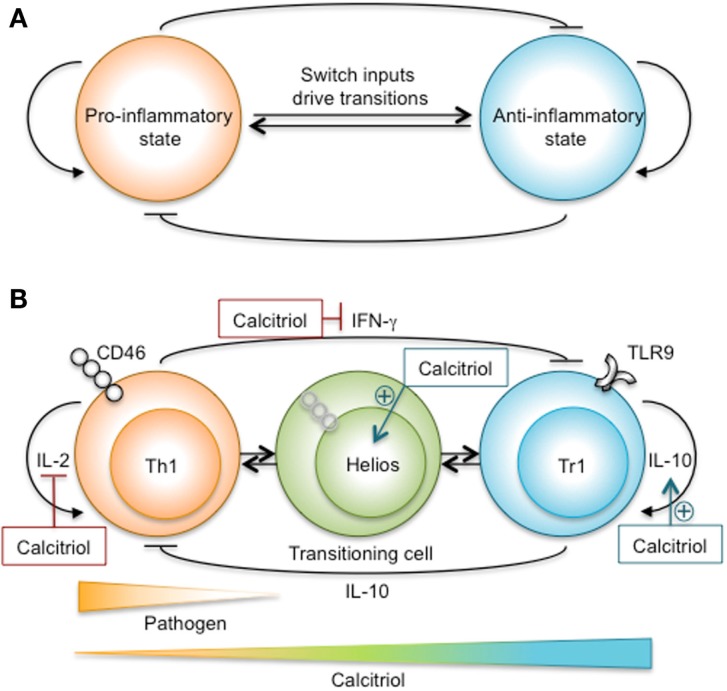
**A proposed Th1–Tr1 bi-stable biological switch bridging two immune states, pro-inflammatory and anti-inflammatory**. **(A)** A theoretical diagram of two opposing immune states [adapted from a generalized bi-stable switch diagram (302)]. Each circle represents T cells that are in a stable biological state characterized by a particular gene regulatory network and capable of responding to external stimuli in a biologically appropriate manner. Biological barriers impede transitions between the pro- and anti-inflammatorystates. Each biological state is capable of reinforcing its own state, denoted as lines terminating in arrows, and inhibiting the opposing state, denoted as blocked lines. External stimuli, for example pathogens, hormones, cell-cell interactions, and soluble mediators serve as switch inputs to drive a transition. For example, signals 1 and 2 can be organized as “1 or 2”, “1 and 2”, or “1 not 2” to trigger a dynamic transition between the two states. **(B)** The proposed Th1–Tr1 switch appears to have the qualities of a bi-stable biological switch. The Th1 cytokine IFN-γ and the Tr1 cytokine IL-10 impede the development of Tr1 and Th1 cells, respectively. The autocrine growth factors IL-2 and IL-10 reinforce the survival and proliferation of Th1 and Tr1 cells, respectively. Diminishing pathogen, sensed through TLR molecule engagement (280), maturation of the pathogen-specific immune response, sensed through CD46 molecule engagement by C3b and C4b (299, 300), and accumulating calcitriol in the microenvironment, sensed through the VDR in Th1 cells, provide the external stimuli to alter the dynamics of the system and drive the transition to the Tr1 biological state. Helios may be a biomarker of effector T cells undergoing a transition to a tolerant state (183). Calcitriol increased Helios in CD4^+^ T cells at the beginning of a calcitriol-induced EAE disease remission (144), and was recently identified as an inhibitor of *Il2* gene transcription by an epigenetic silencing mechanism (184).

Important new research into the function of CD46 has demonstrated the existence of a calcitriol-mediated Th1–Tr1 switch controlling the delicate homeostatic balance between the pro-inflammatory and anti-inflammatory states (Figure 2). CD46 and its murine analog complement receptor 1-related protein Y (Crry) are transmembrane glycoproteins that bind complement fragments C3b and C4b, and function as T-cell costimulatory molecules (269). Temporal processing of two alternatively spliced CD46 cytoplasmic tails, Cyt1 and Cyt2, served as a molecular rheostat controlling activation and de-activation of human T-cell responses (317). Co-engagement of the TCR and CD46 first enhanced Th1 cell effector function (318). However, as IL-2 accumulated, CD46 cytoplasmic domain interactions with the serine–threonine kinase SPAK promoted effector Th1 cell switching to a Tr1 phenotype with low IL-2 production, high IL-10 production, and the capability to suppress bystander T-cell activation. Thus, CD46 served as a molecular link between complement activation and a Th1–Tr1 switch for immune response termination (269). This switch was defective in MS (265, 266), asthma (319), and rheumatoid arthritis patient T cells (318), effectively locking the immune response in an interminable pro-inflammatory state.

Calcitriol profoundly amplified the Th1–Tr1 switch *in vitro* in T cells from healthy donors and patients with MS (219). Calcitriol also amplified this switch *in vitro* in T cells from T1D patients, imprinting the T cells with an IL-10-producing Tr1 phenotype, and modulating surface expression of chemokine receptors to enable homing to inflamed sites (220, 312). Determining whether this switch functions *in vivo* is a high priority goal. The emerging data suggest that *vitamin D may significantly influence the emergence of an autoimmune disease phenotype by controlling a Th1–Tr1 switch that provides a bridge between two apparently stable immune states, pro-inflammatory and anti-inflammatory*. The calcitriol-driven Th1–Tr1 switch is of utmost importance to autoimmune disease, because each state appears to be self-reinforcing and capable of blocking the opposing state (Figure 2). Multistable switches are known to play an important role in hematopoietic cell fate decisions controlled by gene regulatory networks (320), and hormones are known to be of paramount importance in controlling these networks.

Calcitriol enhancement of the transcription factor Helios may also be important to the Th1–Tr1 switch. Calcitriol treatment transiently induced CD4^+^Helios^+^ T cells in the CNS of mice with EAE prior to the loss of pathogenic CD4^+^ T cells from the CNS and the onset of remission (216). New data show Helios was a marker for peripheral T cells that are being driven to tolerance in response to a genuine autoantigen in autoimmune gastritis (321). Helios was identified as a key inhibitor of *Il2* gene transcription in Treg cells (322); it repressed the *Il2* locus through epigenetic modifications that included histone deacetylation.

Given the importance of pathways downstream of calcitriol signaling to the Th1–Tr1 switch and immune response termination, it is valuable to consider how those pathways could be compromised. CD46 is the receptor for the T lymphotropic human herpesvirus-6; the virions bind CD46 and trigger endocytosis (323). Whether pathogen-mediated CD46 removal would undermine calcitriol action by disabling the Th1–Tr1 switch is not known. Genetically determined defects in IL-10 production and IL-10R signaling would be downstream of a calcitriol-mediated increase in Tr1 cells; both defects were linked to an increased autoimmune disease risk (324–329). The IL-10R signals through tyrosine kinase 2 (TYK2) and Janus kinase-1 (330). A novel missense mutation in the *TYK2* gene was linked to an increased risk of MS (329). In addition, recombinant hIL-10 did not inhibit proliferation of CD4^+^ T cells from MS patients; the IL-10R signaling pathway was blocked at the point of STAT3 activation in these cells (267). Defective IL-10R signaling downstream of calcitriol action to induce Tr1 cells could compromise this vitamin D-mediated protective mechanism.

## Conclusion and Future Research Directions

This review article sought to summarize and integrate research on vitamin D and CD4^+^ T-lymphocyte biology in an effort to develop a new understanding of the molecular etiology of autoimmune disease. There is a large, latitude-linked, non-transmissible environmental component that acts at the population level to determine the emergence of an autoimmune phenotype, given an autoimmune disease-susceptible genotype. Diverse and compelling evidence suggests that this environmental component is ambient UVB light exposure catalyzing cutaneous vitamin D_3_ formation. Importantly, this environmental component exerts a >100-fold influence on the risk of multiple autoimmune diseases, and on disparate populations with distinct genotypes, dietary habits, and exposures to infectious and commensal organisms, larger than the influence of the strongest autoimmune susceptibility locus (MHCII genes; approximately fivefold) or the infectious organism, EBV (approximately two to threefold).

The strength and universality of ambient UVB light and vitamin D_3_ as autoimmune disease risk factors, the vitamin D hormone’s established role as a transcriptional regulator of gene expression, and the role of CD4^+^ T lymphocytes as autoimmune disease initiators and suppressors provided the rationale for closely examining calcitriol regulation of CD4^+^ T-lymphocyte function in this review. The conclusions supported by experimental evidence are presented in Box 1. These conclusions address the Bradford Hill criterion of a plausible biological mechanism that coheres with known biological facts (115). This evidence has contributed to a greater functional and mechanistic understanding of the molecular etiology of autoimmune disease, one of the major challenges in modern immunobiology.

Box 1 **Summary of main conclusions regarding vitamin D, CD4^+^ T lymphocytes, and autoimmune disease.**

An autoimmune disease phenotype emerges when modifiable environmental stressors act on a disease-susceptible genotype, and exposure to at least one environmental stressor is increasing.

Vitamin D is probably the environmental factor with the greatest influence on the emergence of an autoimmune disease phenotype given a disease-susceptible genotype.

At high latitudes, there is a period of “light starvation” when vitamin D synthesis is negligible; the higher the latitude, the greater is the light starvation period.

Paracrine calcitriol signaling to CD4^+^ T cells within tissues is likely the major pathway by which sunlight as an environmental factor exerts its influence on the emergence of an autoimmune disease phenotype.

The VDR–RXR complex regulates gene expression in a cell type-specific manner through VDRE in calcitriol-responsive genes.

The hormonal form of vitamin D may influence thymic selection, which would have a significant influence on autoimmune disease risk acquisition and the timing of autoimmune disease prevention initiatives.

The hormonal form of vitamin D may significantly influence autoimmune disease risk and severity by (i) dampening pathogenic Th17 cell IL-17 synthesis, (ii) increasing effector CD4^+^ T-cell sensitivity to extrinsic cell death signals, (iii) promoting CD4^+^CD25^+^FoxP3^+^ Treg cell and CD4^+^IL-10^+^FoxP3^−^ Tr1 cell development and suppressive function, (iv) amplifying a Th1–Tr1 switch that may bridge two apparently stable immune states, an anti-pathogen pro-inflammatory state and a self-tolerant anti-inflammatory state.

During periods of light starvation, phototherapy to increase FoxP3^+^ Treg cell development may be a particularly efficient method of influencing autoimmune disease risk and severity.

Important questions remain. Regarding paracrine calcitriol signaling to CD4^+^ T cells, more work is needed *in vivo* to understand the cellular sources of calcitriol, the signals and kinetics that induce and terminate calcitriol synthesis and their relationship to infectious organisms. More work is also needed to understand the signals and kinetics that induce and terminate *VDR* and *CYP24A1* gene expression in specific types of CD4^+^ T cells *in vivo*, and how gender influences calcitriol responsiveness and calcitriol turnover. Finally, we must learn what factors and mechanisms disrupt paracrine signaling (genetic lesions, drugs, oxidative damage to enzymes, epigenetic dysregulation).

A second and related high priority is probing a possible vitamin D influence on thymic selection. Such an influence would have high significance for our understanding of the molecular etiology of autoimmune disease risk acquisition and our effort to correctly time disease prevention efforts. The data correlating seasonal variation in thymic output inversely with vitamin D status await replication. Assuming reproducibility, mechanistic inquiry will be needed to distinguish potential impacts on thymopoiesis and positive selection from negative selection, and to decipher the calcitriol-regulated genes that influence these processes.

Other questions relate to calcitriol actions on effector CD4^+^ T cells *in vivo*. Calcitriol suppression of *Il17* transcription and IL-17 protein synthesis has been studied mechanistically in rodents with conflicting results. It will be important to clarify the biochemistry of *Il17* transcriptional and post-transcriptional regulation by calcitriol in Th17 cells *in vivo* in the context of different autoimmune disease settings where physiologically relevant tissue architecture and cell and cytokine microenvironments exist. Other open questions concern the biochemistry of the effector CD4^+^ T-cell programed cell death pathways that are influenced by calcitriol, and the cellular source and timing of the extrinsic cell death signaling.

Another very high priority is probing how vitamin D promotes CD4^+^FoxP3^+^IL-10^−^ Treg and CD4^+^FoxP3^−^IL-10^+^ Tr1 cell development, stability, and suppressive function. Unanswered questions relate to the biochemical mechanisms for the proposed calcitriol up-regulation of *Ikzf2*, *Il10*, *Ctla4*, and *CD200* gene transcription, and the relationship between *VDR* and *FOXP3*, how each gene may impact the other’s expression and the kinetics and stability of the interaction.

Additional questions concern the molecular details of the proposed Th1–Tr1 switch. Whether this switch functions *in vivo* in the context of different autoimmune diseases is a top priority question. Moreover, questions remain regarding the precise biochemical details of the switch, the signals that trigger it, how the signals are transduced to the nucleus, how the gene expression program is altered by those signals, and exactly how calcitriol amplifies the switch. This molecular knowledge is needed to envision and probe how pathogen-mediated engagement of CD46 might compromise the switch. Mathematical modeling of the switch may be helpful to envision interactions between environmental pathogens, the sun-sensitive hormone, the pathogen-specific immune response, immune response termination, and self-tolerance maintenance.

Sir Austin Bradford Hill suggested minimal criteria for judging the causal nature of relationships between environmental factors and disease (115). Many experts believe the experimental evidence linking vitamin D_3_ with autoimmune disease risk is sufficiently compelling to satisfy eight of the nine criteria (6). Perhaps, the most important unanswered question derives from Bradford Hill’s ninth criterion, experiment. Would an intervention that substantially elevates circulating 25-OHD_3_ prevent or change autoimmune disease in light-starved populations with low vitamin D status? Now would be a good time for autoimmune disease researchers to address this unanswered question collaboratively.

## Conflict of Interest Statement

The authors declare that the research was conducted in the absence of any commercial or financial relationships that could be construed as a potential conflict of interest.

## Abbreviations

25-OHD_3_, 25-hydroxyvitamin D_3_; APC, antigen-presenting cells; calcitriol, 1α,25-dihydroxyvitamin D_3_; CNS, central nervous system; CTLA4, cytotoxic T-lymphocyte antigen 4; DC, dendritic cell; EAE, experimental autoimmune encephalomyelitis; EAU, experimental autoimmune uveitis; EBV, Epstein-Barr virus; IFN, interferon; IL, interleukin; MHC, major histocompatibility complex; MRI, magnetic resonance imaging; MS, multiple sclerosis; NOD, non-obese diabetic; RANK, receptor-activator of NF-kappaB; RANKL, receptor-activator of NF-kappaB ligand; T1D, type 1 diabetes; TCR, T-cell receptor; Th, T helper; TLR, Toll-like receptor; Tr1, T-regulatory cell type 1; Treg, T-regulatory; TYK2, tyrosine kinase 2; UV, ultraviolet; VDR, vitamin D receptor; VDRE, vitamin D response element.
